# Four-Dimensional Flow MRI of Abdominal Veins: A Systematic Review

**DOI:** 10.3390/diagnostics11050767

**Published:** 2021-04-24

**Authors:** Simon O. Haarbye, Michael B. Nielsen, Adam E. Hansen, Carsten A. Lauridsen

**Affiliations:** 1 Department of Diagnostic Radiology, Rigshospitalet, Copenhagen University Hospital, DK-2100 Copenhagen, Denmark; mbn@dadlnet.dk (M.B.N.); Adam.espe.hansen@regionh.dk (A.E.H.); cala@phmetropol.dk (C.A.L.); 2 Department of Technology, Faculty of Health and Technology, Metropolitan University College, DK-2100 Copenhagen, Denmark; 3 Department of Clinical Medicine, University of Copenhagen, DK-1165 Copenhagen, Denmark

**Keywords:** 4D MRI, diagnostics, abdominal veins, scan techniques, hemodynamics

## Abstract

The aim of this systematic review is to provide an overview of the use of Four-Dimensional Magnetic Resonance Imaging of vector blood flow (4D Flow MRI) in the abdominal veins. This study was composed according to the PRISMA guidelines 2009. The literature search was conducted in MEDLINE, Cochrane Library, EMBASE, and Web of Science. Quality assessment of the included studies was performed using the QUADAS-2 tool. The initial search yielded 781 studies and 21 studies were included. All studies successfully applied 4D Flow MRI in abdominal veins. Four-Dimensional Flow MRI was capable of discerning between healthy subjects and patients with cirrhosis and/or portal hypertension. The visual quality and inter-observer agreement of 4D Flow MRI were rated as excellent and good to excellent, respectively, and the studies utilized several different MRI data sampling strategies. By applying spiral sampling with compressed sensing to 4D Flow MRI, the blood flow of several abdominal veins could be imaged simultaneously in 18–25 s, without a significant loss of visual quality. Four-Dimensional Flow MRI might be a useful alternative to Doppler sonography for the diagnosis of cirrhosis and portal hypertension. Further clinical studies need to establish consensus regarding MRI sampling strategies in patients and healthy subjects.

## 1. Introduction

Phase contrast Magnetic Resonance Imaging (MRI) is widely used for the clinical evaluation of blood flow in the heart and in large vessels [1]. Developments of the technique have enabled the measurement of time-resolved vector blood flow in three anatomic dimensions, also known as 4D Flow MRI. Studies have shown that 4D Flow MRI can provide full, velocity encoded, volumetric coverage of the heart, aorta and thoracic arteries [2,3,4,5,6,7]. This allows for the acquisition of quantitative hemodynamic parameters such as blood flow velocity and blood flow volume, while simultaneously visualizing the direction and velocity of blood flow as velocity vectors, by using streamlines and particle tracing. To achieve this, 4D Flow MRI uses the phase contrast technique, in which bipolar magnetic field gradients create a phase shift of the MR signal proportional to the flow velocity. The sensitivity of phase contrast MRI to flow velocities is controlled by the velocity encoding (venc) parameter, which must be set to the expected maximum flow velocity. After the acquisition of phase contrast MRI, flow visualization software is used to create volumetric blood flow images. [8]. The ability to combine hemodynamic data with visual assessment in a single scan sequence may permit the exploration of diagnostic markers in any vascular region of interest. As with other MRI scanning techniques, 4D Flow MRI can use different sampling methods, which may affect the scan time and image quality [9,10,11]. The results from several studies indicate that the hemodynamic parameters acquired by 4D Flow MRI have the potential to reveal arterial pathologies such as aortic stenosis and chronic obstructive pulmonary disease [3,6,12].

The utility of 4D Flow MRI in the abdominal area and for the venous system is less explored, as compared to cardiovascular applications. Even so, some studies have investigated 4D Flow MRI as a diagnostic tool for pathologies in the abdominal venous vasculature, such as cirrhosis and portal hypertension [13,14].

These pathologies can already be examined non-invasively by Doppler sonography. Though Doppler has a short examination time for single blood vessels [15], 4D Flow MRI may acquire volumetric flow data in several blood vessels simultaneously, and thus 4D Flow MRI may have a greater clinical applicability for the evaluation of pathologies of abdominal veins.

To the knowledge of the authors, no systematic review of 4D Flow studies outside the cardiovascular system has been published. The purpose of this systematic review is to create an overview of the published literature evaluating the feasibility of 4D Flow MRI as a diagnostic tool in abdominal veins.

## 2. Materials and Methods

### 2.1. Search Strategy

The eligibility criteria and analysis in this review were performed according to the PRISMA guidelines 2009 (Preferred Reporting Items for Systematic Reviews and Meta-Analyses) [16]. The literature search was conducted in the following databases: MEDLINE, Cochrane Library, EMBASE, and Web of Science. The intention of the search was to identify studies applying 4D Flow MRI in abdominal veins. Selection criteria for inclusion in this review were studies with human subjects, published in or after 2010, written in English, including implementation of 4D Flow MRI in the abdominal veins. The last search was performed on the 22 February 2021. Since the use of free text was different in the applied databases, it was necessary to tailor the search for each database. It should be noted that at the time of the search and of writing, 4D Flow MRI has not yet been made a MeSH term. Therefore, the search string required more free text to reduce the risk of missing relevant studies. The final search string can be found in Appendix A.

### 2.2. Study Selection

**Figure 1:**
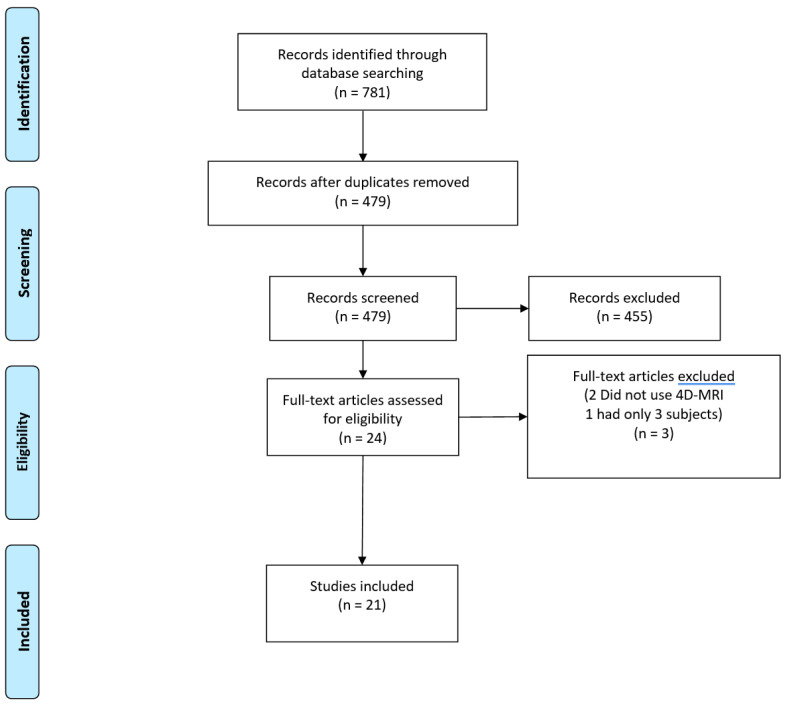
PRISMA flowchart of the inclusion process.

The included studies were filtered for duplicates by using Covidence, a web-based systematic review software. Two authors with 20 years and 1 year of experience in radiography (C.L. and S.H., respectively) reviewed the relevance of the studies, starting with the relevance of the title, then the relevance of the abstract and then the relevance of the full text. Disagreement on relevance and inclusion was resolved in consensus. As shown on the PRISMA flowchart of the inclusion process in Figure 1, the literature search yielded 781 publications. Of the yielded publications, 302 were duplicates and 455 were deemed irrelevant, on account of not applying 4D Flow MRI or not applying 4D Flow MRI in abdominal veins. After full-text assessment, three additional studies were deemed ineligible. Two did not include use of 4D Flow MRI, one had only 3 subjects.

### 2.3. Quality Assessment

Lastly, the Quality Assessment of Diagnostic Accuracy Studies (QUADAS-2) tool was used to assess the risk of bias and applicability in the included studies [17]. Risks of bias and applicability were classified as high, low, or unclear by the same two authors who selected studies for inclusion.

## 3. Results

### 3.1. Overview of Included Studies

A table (Table 1) of relevant data in each study was made using the following headings: author and publication year, aim of study, number of subjects, sampling methods, scan time, range of blood flow velocities for velocity encoding (venc), examined venous structures, and conclusion of study.

### 3.2. Anatomic Coverage of Included Studies

All the included studies successfully applied 4D Flow MRI in abdominal veins. Vascular structures imaged in each study can be seen in Table 2.

### 3.3. Subject and Patient Types in Included Studies

Twelve studies included healthy subjects [14,18,26]. Ten studies included patients with cirrhosis and/or portal hypertension [1,10,13,14,18,19,20,21,27,28]. One study included patients with chronic liver disease [10]. One study included pediatric patients with either a non-operated portal venous system or with a surgically created portal shunt [29]. One study included patients with cirrhosis and varices [28]. One study included patients with hepatitis and nonalcoholic steatohepatitis [22]. Three studies included patients with transjugular intrahepatic portosystemic shunts [30,32,34].

Seven studies displayed that 4D Flow MRI was capable of discerning between healthy subjects and patients with cirrhosis and/or portal hypertension [1,10,13,18,19,20,21].

### 3.4. Visual Quality, Inter-Observer Agreement, Sampling Method, and Scan Times

Nine out of 21 studies analyzed the visual quality of the acquired 4D Flow MRI scans [13,18,19,20,23,24,27,29,30]. The nine studies reported a good to very good or excellent visual quality, though the left portal vein branch was reported to have a lower visual quality than other scanned veins in six of the seven studies that documented the visual quality in this vein [13,18,20,23,24,30]. Seven studies assessed inter-observer agreement. Five studies rated the inter-observer agreement as substantial, high or excellent [1,10,13,20,24,31]. One study rated it as good [18].

Of all 21 included studies, two studies used a cartesian sampling method [1,29], nine used a radial sampling method [14,19,21,25,26,27,28,32], and one used a spiral sampling method with compressed sensing [10]. One study performed cartesian sampling and spiral sampling with compressed sensing in succession [22]. Nine studies did not state details of the MRI sampling method [13,18,20,23,24,30,31,33,34]. In addition to the sampling method, four studies also used k-t GRAPPA (Generalized Autocalibrating Partially Parallel Acquisitions) [18,23,29,31].

The studies that applied cartesian sampling had a scan time of 6 to 15 min [1,22,29]. Eight of the studies with applied radial sampling had a scan time of 10 to 12 min [14,21,26,27,28,32], and one that applied time averaging had a scan time of down to three to four minutes [19]. The two studies with spiral sampling and compressed sensing applied had a scan time from 18 to 25 s [10,22].

### 3.5. Applicability and Risk of Bias

The QUADAS-2 assessment on risk of bias and concerns about applicability can be seen in Table 3 below. All studies were considered to have an overall low risk of bias, though several studies had an unclear risk in the index test and reference standard.

Risk of bias in patient selection was considered high if the examined study did not make use of consecutively or randomly selected subjects, used a case–control design, or made inappropriate exclusions.

Risk of bias in the index test was considered high if the examined study interpreted the index test results with knowledge of the reference standard.

Risk of bias in the reference standard was considered high if the examined study interpreted the results of the reference standard with knowledge of the results the index test or used a reference standard that was unlikely to correctly classify the target condition. Risk of bias in flow and timing was considered high if the examined study had an inappropriate time interval between the index test and the reference standard, did not include all its subjects in the analysis or did not use the same reference standard for all its subjects. Studies considered to have an unclear risk of bias in the index test and reference standard did not state whether they interpreted their results without knowledge of the reference standard and did not state whether they interpreted the results of the reference standard without knowledge of the index test. Studies with an unclear risk of bias in flow and timing did not state the time interval of their data acquisition.

## 4. Discussion

In summary, the results from the included studies show that 4D Flow MRI of the abdominal veins is feasible, and that hemodynamic parameters based on 4D Flow MRI can be used to discern between healthy subjects and patients with cirrhosis and/or portal hypertension. The visual quality of the acquired flow images was rated good to very good and the included studies indicate that 4D Flow MRI has a low inter-observer variability.

The studies included in this systematic review were considered to have low bias.

The acquisition time of spiral sampling combined with compressed sensing was remarkably faster (18 to 25 s) than the cartesian and radial sampling methods (6 to 15 min and 3 to 12 min, respectively) [1,10,14,19,21,22,26,27,28,29,32]. The acceleration method known as k-t GRAPPA was successfully applied in four studies, though the acquisition times of these studies were still slower than the studies that applied spiral sampling and compressed sensing [10,18,22,23,29,31].

There is no significant loss of visual quality when applying spiral sampling to 4D Flow MRI in the aorta, despite the major reduction in scan time it provides, according to the included studies [10,22]. Compressed sensing has been stated to have no significant effect on visual quality when not overused; however, it does increase computational complexity, thereby potentially increasing the reconstruction time of the acquired image data [35]. The two included studies which combined spiral sampling and compressed sensing demonstrated that 4D Flow MRI with spiral sampling and compressed sensing can be applied in the abdominal veins, with greatly reduced scan time compared to radial sampling with or without k-t GRAPPA and cartesian sampling, and still maintain a good vascular conspicuity and strong agreement of the acquired quantitative parameters with those from established techniques [10,22].

Doppler sonography’s high inter-observer variability and limited ability to visualize complex and variable anatomy in the abdominal vasculature compared to 4D Flow MRI were demonstrated by several studies included in this systematic review [10,13,18,20,23,24,27,29,30,31]. This assessment seems to agree with the established literature. According to the consensus statement of P. Dyverfeldt et al. [36], volumetric flow imaging of 4D Flow MRI may provide a more accurate flow quantification in the presence of complex vessel geometry. In addition to this, included studies showed that the ability of 4D Flow MRI to assess volumetric flow in multiple veins simultaneously could potentially provide a better overview of blood flow parameters in the abdominal veins compared to Doppler sonography, in which each vein would have to be examined individually. Therefore, 4D Flow MRI may have potential as an alternative to Doppler sonography for the non-invasive measurements of hemodynamic parameters in the abdominal venous vasculature.

While the included studies indicate that 4D Flow MRI with spiral sampling and compressed sensing had a higher inter-observer agreement than Doppler sonography, one should also consider the comparatively high cost that is associated with MRI [37,38]. In addition to this, while 4D Flow MRI with spiral sampling and compressed sensing has a remarkably short acquisition time, Doppler sonography does not require the same amount of time for patient preparation and MRI safety as 4D Flow MRI. This means that the full examination time of 4D Flow MRI with spiral sampling and compressed sensing may still be notably higher than the examination time of Doppler sonography.

This study had limitations. Since 4D Flow MRI did not have a MeSH term at the time of the literature search, and the scan technique has several different proposed names, it is possible that some studies on 4D Flow MRI were not found during the literature search. The evaluation on how the choice of sampling method affects the scan time and visual quality of 4D Flow MRI was limited due to nine studies not stating their applied sampling method [13,18,20,23,24,30,31,33,34]. The limited number of participants (from 3 to 61) and the different patient groups were heterogenous and, therefore, it was not possible to make a collective conclusion of the expected hemodynamic parameters derived from 4D Flow MRI. For example, the 11 studies that investigated 4D Flow MRI’s application in patients with cirrhosis had variations in patient age and severity of cirrhosis [1,10,13,18,20,25,27,28,30,31,33]. This also limited the possibility of making an estimate of the recommended venc value for future studies.

Repetition of the included studies with larger subject/patient groups is recommended to assess the possibility of hemodynamic data acquired by 4D Flow MRI as a diagnostic marker for cirrhosis or portal hypertension. In addition, this systematic review suggests that future research on 4D Flow MRI should use spiral sampling with compressed sensing, as it appears to be the most clinically viable path for 4D Flow MRI, and should further assess the reliability of this sampling method.

## 5. Conclusions

In conclusion, 4D Flow MRI examination of abdominal veins for the purpose of visual assessment and quantification of hemodynamic parameters is feasible. The hemodynamic parameters derived from 4D Flow MRI can be used to discern between healthy subjects and patients with cirrhosis and/or portal hypertension. Four-Dimensional Flow MRI of the abdominal veins has a higher inter-observer agreement than Doppler sonography, which is a currently used non-invasive method, and permits the acquisition of flow data in several vessels simultaneously. Recent developments of MRI sampling methods have allowed 4D Flow MRI scans of the abdominal veins to be acquired in approximately 20 s, while still maintaining good vessel conspicuity and reliability of hemodynamic data, thus potentially greatly facilitating routine clinical use.

## Figures and Tables

**Table 1:** Aims, subject types, scan parameters, scanned veins, and conclusions of included studies.

Author, Year	Aims	Human Subjects		Scan Parameters			Scanned Veins	Conclusion
				Sampling Method	Scan Time Healthy Subjects/Patients (Minutes)	Venc (cm/s)		
Brunsing et al., 2021 [1]	To determine the feasibility of quantifying the hemodynamic effects of cirrhosis with 4D Flow MRI, and introduce hydraulic circuit indexes of severity.	21 Patients without known liver disease	26 Cirrhosis patients	Cartesian	6–15/6–15	80–150	Portal vein	Quantification with 4D Flow MRI was technically feasible and showed promise in quantifying the hemodynamic effects of cirrhosis. Proposed quantitative metrics of hepatic vascular resistance correlated with PSS.
Bane et al., 2019 [10]	To determine the reproducibility of flow quantification in abdominal vessels using 4D MRI. To assess the value of 4D Flow MRI parameters in diagnosing cirrhosis and degree of portal hypertension.	52 Chronic liver disease patients		Spiral with compressed sensing	N/A/22 s	60	Superior mesenteric vein Splenic vein Portal vein Suprarenal inferior vena cava Infrarenal inferior vena cava Right hepatic vein Middle hepatic vein	4D MRI using spiral sampling allows comprehensive assessment of abdominal vessels in one breath-hold, with substantial inter-observer reproducibility. 4D MRI may potentially reflect vascular changes due to cirrhosis and portal hypertension.
Stankovic et al., 2012 [13]	To compare 4D MRI with Doppler US’ ability to display hemodynamics in the portal venous system of cirrhotic patients and healthy controls.	41 Healthy subjects	20 Cirrhosis patients	N/A	18.1 to 21.9/14.6	50	Superior mesenteric vein Splenic vein Splenic Mesenteric Confluence Right Portal vein branch Left Portal vein branch	4D MRI may constitute an alternative technique to Doppler US for evaluating hemodynamics in the portal venous system of patients with cirrhosis.
Roldán-Alzate et al., 2013 [14]	To validate 4D MRI for quantification of blood flow in the portal venous system of healthy volunteers and patients with portal hypertension.	7 Healthy subjects	17 Portal hypertension patients	Radial	10 to 12/10 to 12	60	Superior mesenteric vein Splenic vein Portal vein Right Portal vein branch Left Portal vein branch	Quantification of blood flow in the portal venous system of patients with portal hypertension using 4D MRI is very feasible.
Stankovic et al., 2010 [18]	To evaluate the feasibility of 4D MRI visualization of the portal venous system’s hemodynamics.	18 Healthy subjects	5 Cirrhosis patients	k-t GRAPPA	22.46/16.40	50	Superior mesenteric vein Splenic vein Splenic Mesenteric Confluence Right Portal vein branch Left Portal vein branch	4D MRI is feasible for visualization of the portal venous systems hemodynamics.
Landgraf et al., 2014 [19]	To reduce the scan time of radial 4D Flow MRI by using temporal averaging.	15 Healthy subjects	29 Cirrhosis patients	Radial	3–4/3–4	60	Superior mesenteric vein Splenic vein Portal vein	The scan time of radial 4D Flow MRI may be reduced by 50–75% by means of time averaging.
Stankovic et al., 2013 [20]	To evaluate the feasibility of 4D MRI for the display of hemodynamics in the portal venous system of patients with cirrhosis and healthy controls.	10 Healthy subjects	5 Cirrhosis patients	N/A	12.4/13.9	100	Superior mesenteric vein Splenic vein Splenic Mesenteric Confluence Right Portal vein branch Left Portal vein branch	4D MRI is feasible for profound evaluation of the portal venous systems hemodynamics in patients with cirrhosis and healthy controls.
Roldán-Alzate et al., 2015 [21]	To quantify changes in abdominal hemodynamics in patients with portal hypertension undergoing meal challenge using 4D MRI.	6 Healthy subjects	12 Portal hypertension patients	Radial	12/12	Pre meal = 100 post meal = 120	Superior mesenteric vein Splenic vein	Regulation of flow in the portal venous system after a meal challenge may be impaired in patients with cirrhosis.
Dyvorne et al., 2015 [22]	To develop a highly accelerated phase-contrast cardiac-gated hemodynamic measurement 4D MRI technique based on spiral sampling and dynamic compressed sensing. To compare this technique with cartesian sampling imaging techniques for the quantification of flow in abdominal vessels.	3 Healthy subjects	7 liver disease patients	Cartesian and Spiral with compressed sensing in two separate acquisitions	Cartesian:11.4/11.4 Spiral sampling with compressed sensing: 18 to 25 s /18 to 25 s	60	Superior mesenteric vein Splenic vein Portal vein Suprarenal inferior vena cava Infrarenal inferior vena cava Right hepatic vein Middle hepatic vein Left hepatic vein Right renal vein Left renal vein	The combination of spiral sampling with dynamic compressed sensing results in major acceleration for 4D MRI and allows assessment of abdominal vessel hemodynamics in a single breath hold. Good vascular conspicuity was observed in abdominal vessels, although with decreased image quality. Quantitative parameters were in strong agreement with cartesian sampling techniques
Stankovic, Fink et al., 2015 [23]	To evaluate the feasibility of using k-t GRAPPA to accelerate 4D MRI in the portal venous system by investigating the impact of different acceleration factors.	16 Healthy subjects		k-t GRAPPA	4.0 to 13.9/N/A	100	Superior mesenteric vein Splenic vein Splenic Mesenteric Confluence Right Portal vein branch Left Portal vein branch	k-t GRAPPA-accelerated 4D MRI assessment of the portal venous systems hemodynamics is feasible while achieving a significant reduction in scan time.
Stankovic et al., 2014 [24]	To evaluate the effect of variation in different spatio-temporal resolutions and reproducibility in portal venous 4D MRI.	10 Healthy subjects		N/A	8.2 to 14.6/N/A	100	Superior mesenteric vein Splenic vein Splenic Mesenteric Confluence Right Portal vein branch Left Portal vein branch	Higher spatio-temporal resolution is necessary for complete assessment of hemodynamics required for clinical applications. Four-dimensional MRI can be performed with good reproducibility.
Rutkowski et al., 2019 [25]	To examine the effects of varying superior mesenteric confluence anatomy on hemodynamics in the portal venous system.	9 Healthy subjects	6 Cirrhosis patients	Radial	N/A	N/A	Superior mesenteric vein Splenic vein Portal vein Right Portal vein branch Left Portal vein branch	There was significant correlation between vessel anatomy and hemodynamics.
Roberts et al., 2021 [26]	To assess the feasibility of quantitatively evaluating mesenteric hemodynamics before and after a meal challenge in patients suspected of having chronic mesenteric ischemia (CMI) and healthy controls.	20 Healthy subjects	19 Patients with suspected CMI	Radial	11/11	Pre meal = 100 post meal = 120	Superior mesenteric vein Splenic vein Portal vein	4D Flow MRI demonstrated significant differences in the redistribution of blood flow in the scanned vessels of CMI positive patients after a meal challenge.
Frydrychowicz et al., 2011 [27]	To assess the feasibility of using 4D MRI to display hemodynamics of the portal venous system in patients with portal hypertension.	24 Cirrhotic patients		Radial	10 to 12/10 to 12	60	Superior mesenteric vein Inferior mesenteric vein Splenic vein Portal vein Right Portal vein branch Left Portal vein branch	4D MRI provides a comprehensive, volumetric approach to assess hemodynamics of the portal venous system in patients with portal hypertension.
Motosugi et al., 2019 [28]	To assess the feasibility of 4D MRI as a noninvasive imaging marker to classify the risk of variceal bleeding in patients with cirrhosis.	8 Cirrhosis patients without varices	15 Cirrhosis patients with varices	Radial	N/A/10	30	Superior mesenteric vein Splenic vein Portal vein Azygos vein	4D MRI data of the azygos, splenic, superior mesenteric and portal venous flow are useful markers to classify the risk of variceal bleeding in patients with cirrhosis.
Parekh et al., 2017 [29]	To assess the feasibility of 4D MRI’s ability to visualize portal venous hemodynamics in children and young adults.	28 pediatric patients		Cartesian With k-t GRAPPA	8 to 10/8 to 10	40–80	Superior mesenteric vein Splenic vein Portal vein Right hepatic vein Middle hepatic vein Left hepatic vein Inferior vena cava	4D MRI is feasible for the 3D visualization of portal venous hemodynamics in children and young adults.
Stankovic, Rössle et al., 2015 [30]	To assess changes in portal hemodynamics in patients undergoing transjugular intrahepatic portosystemic shunt (TIPS) using 4D MRI.	11 Cirrhosis patients		N/A	N/A/10 to 20	225	Only transjugular intrahepatic portosystemic shunts	4D MRI can detect TIPS patency and stenosis, but further investigation is required before it can be used to assess for TIPS dysfunction.
Keller, Collins et al., 2017 [31]	To compare an alternative preprocessing workflow to a conventional workflow in abdominal 4D MRI.	20 Patients with cirrhosis and portal hypertension		k-t GRAPPA	N/A/15	50	Superior mesenteric vein Splenic vein Portal vein Right Portal vein branch Left Portal vein branch Right hepatic vein Middle hepatic vein Left hepatic vein	Superior abdominal 4D MRI data consistency was obtained by applying an alternative preprocessing workflow.
Bannas et al., 2016 [32]	To assess the feasibility of 4D MRI monitoring of portal hemodynamics before and after transjugular intrahepatic portosystemic shunt (TIPS) placement.	7 Patients with portal hypertension		Radial	N/A/12	Pre-tips:60 Post-tips: 80 and 120 in two separate scans	Superior mesenteric vein Splenic vein Portal vein	4D MRI is feasible for monitoring of portal hemodynamics before and after TIPS placement.
Keller et al., 2017 [33]	To evaluate the ability of spleen volume, blood flow, and an index incorporating multiple measures, to predict cirrhosis-associated hypersplenism.	39 Patients with cirrhosis and portal hypertension		N/A	N/A	50, 100 and dual-venc (56 and 120) in three separate scans	Superior mesenteric vein Splenic vein Portal vein Right Portal vein branch Left Portal vein branch	A splenic flow index that incorporates both splenic volume and blood flow is a better indicator of hypersplenism than splenic volume alone.
Owen et al., 2018 [34]	To assess the feasibility of detecting patency, stenosis, or occlusion of transjugular intrahepatic portosystemic shunt (TIPS) with 4D MRI.	23 Patients with prior placement of a transjugular intrahepatic shunt		N/A	N/A/10 to 20	225	Only transjugular intrahepatic portosystemic shunts	4D MRI can detect TIPS patency and stenosis, but further investigation is required before it can be used to assess for TIPS dysfunction.

**Table 2:** Veins in which 4D Flow MRI was applied and the number of studies that did so in each vein.

Veins and Structures	Number of Studies
Portal vein	15
Left and right portal vein branches	11
Superior mesenteric vein	20
Inferior mesenteric vein	1
Splenic vein	20
Splenic mesenteric confluence	5
Right hepatic vein	4
Middle hepatic vein	3
Left hepatic vein	4
Azygos vein	1
Transjugular intrahepatic portosystemic shunt	3

**Table 3:** Results from the QUADAS-2 examination of included studies. Risk of bias and concerns regarding applicability of the included studies: ☺ Low Risk; ☹ High Risk; ? Unclear Risk.

Study	Risk of Bias				Applicability Concerns		
	Patient Selection	Index Test	Reference Standard	Flow and Timing	Patient Selection	Index Test	Reference Standard
Brunsing et al., 2021 [1]	☺	?	?	☺	☺	☺	☺
Bane et al., 2019 [10]	☹	?	?	?	☺	☺	?
Stankovic et al., 2012 [13]	☺	☺	☺	☺	☺	☺	☺
Roldán-Alzate et al., 2013 [14]	☺	?	?	☺	☺	☺	☺
Stankovic et al., 2010 [18]	☹	?	☺	☺	☺	☺	☺
Landgraf et al., 2014 [19]	☺	☺	☺	☺	☺	☺	☺
Stankovic et al., 2013 [20]	☺	☺	☺	☺	☺	☺	☺
Roldán-Alzate et al., 2015 [21]	☺	?	?	?	☺	☺	?
Dyvorne et al., 2015 [22]	☺	?	?	?	☺	☺	?
Stankovic, Fink et al., 2015 [23]	☺	?	?	?	☺	☺	?
Stankovic et al., 2014 [24]	☺	?	?	?	☺	☺	?
Rutkowski et al., 2019 [25]	☺	?	?	?	☺	☹	?
Roberts et al., 2021 [26]	☺	?	?	☹	☺	☺	?
Frydrychowicz et al., 2011 [27]	☺	?	?	☺	☺	☺	☺
Motosugi et al., 2019 [28]	☺	?	?	?	☺	☺	?
Parekh et al., 2017 [29]	☺	?	?	?	☺	☺	?
Stankovic, Rössle et al., 2015 [30]	☺	?	?	?	☺	☺	?
Keller, Collins et al., 2017 [31]	☺	☺	☺	☺	☺	☺	☺
Bannas et al., 2016 [32]	☺	?	?	?	☺	☺	?
Keller, Kulik et al., 2017 [33]	☺	?	?	?	☺	☹	?
Owen et al., 2018 [34]	☺	☺	☺	☺	☺	☺	☺

## Data Availability

No new data were created or analyzed in this study. Data sharing is not applicable to this article.

## Abbreviations

4D Flow MRI	Four-Dimensional Magnetic Resonance Imaging of vector blood flow
MRI	Magnetic Resonance Imaging
PRISMA	Preferred Reporting Items for Systematic Reviews and Meta-Analyses
Venc	velocity encoding sensitivity
CMI	chronic mesenteric ischemia
TIPS	transjugular intrahepatic shunt
k-t GRAPPA	Generalized Autocalibrating Partially Parallel Acquisitions

## Appendix A

The following MEDLINE search string was applied in the search for relevant studies in this systematic review. The first section of the search string was intended to find studies that included 4D Flow MRI. The second section was intended to broaden the amount of studies found that included 4D Flow MRI, since not all studies used the same name for 4D Flow MRI. The third section was intended to limit the search result to studies that mentioned the abdominal veins:

“((4D velocity mapping or 4D Flow MR or 4DMRI or 4DMR or MR 4D flow or MRI 4D flow or 4D flow MRI or 4D blood flow MRI or 4D blood flow MR or 4D magnetic resonance or magnetic resonance 4D or 4 dimensional magnetic resonance or 4 dimensional MR or 4D flow magnetic resonance or magnetic resonance 4D flow or 4 dimensional flow magnetic resonance or 4 dimensional flow mri or 4D-Flow MR or MR 4D-flow or MRI 4D-flow or 4D-flow MRI or 4D-magnetic resonance or magnetic resonance 4D or 4-dimensional magnetic resonance or 4-dimensional MR or 4D-flow magnetic resonance or magnetic resonance 4D-flow or 4-dimensional flow magnetic resonance or 4-dimensional flow MR or 4-dimensional-flow magnetic resonance or 4-dimensional-flow MR or 4D phase contrast or 4-dimensional phase contrast magnetic resonance or 4D PC MRI or 4D PC MR or 4D PCMR or 4D PCMRI or four-dimensional phase contrast magnetic resonance or time resolved PC-MRI or time resolved phase contrast magnetic resonance or velocity encoded 4D MRI or velocity encoded 4D MR or 4D MRI flow or flow-sensitive magnetic resonance or phase contrast magnetic resonance with three directional velocity encod* or phase contrast magnetic resonance imaging with three directional velocity encod* or MRI velocity mapping or mr velocity mapping or magnetic resonance velocity mapping or magnetic resonance imaging velocity mapping or MRI velocity encod* or MR velocity encod* or magnetic resonance velocity encod* or magnetic resonance imaging velocity encod* or TWIST MR or TWIST MRI or TWIST Magnetic resonance or TWIST Imaging or TRICKS MR or TRICKS MRI or TRICKS Magnetic resonance or TRICKS Imaging or 4D-TRAK or TRAK MR or TRAK MRI or TRAK magnetic resonance or TRAK imaging or TRAQ or PC VIPR or PC-VIPR or Phase Contrast Vastly undersampled Imaging with Projection Reconstruction or four-dimensional velocity mapping or 4D flow imaging).mp. or ((Magnetic Resonance Angiography/or Magnetic Resonance Imaging/or Magnetic Resonance venography.mp. or MR venography.mp. or MRI venography.mp. or phase-contrast magnetic resonance.mp.) and (4D or 4D or four dimensional or 4 dimensional or velocity encod* or velocity mapping or velocity map or three directional velocity or three dimensional hemodynamic visualization or three directional flow visualization or three directional blood flow visualization or time resolved three directional).mp.)) and (abdomen/or azygos vein/or hepatic veins/or portal system/or renal veins/or abdominal vein.mp. or abdominal veins.mp. or (azygos vein or mesenteric vein, splenic vein, renal vein).mp. or hepatic vein.mp. or Hepatic Veins/or vena porta.mp. or Portal Vein/or portal vein.mp. or (vein or veins).mp. or Veins/or portal venous flow.mp. or Portal venous.mp. or Portal venous hemodynamics.mp. or Portal hemodynamics.mp. or Portal flow.mp. or splanchnic vein.mp. or splanchnic.mp. or azygos venous.mp. or hepatic venous.mp. or renal venous.mp. or abdominal venous.mp. or mesenteric venous.mp. or splenic venous.mp. or portal hypertension.mp. or cirrhotic.mp. or hepatic.mp.)”.
